# Quantitative, model-based estimates of variability in the generation and serial intervals of *Plasmodium falciparum* malaria

**DOI:** 10.1186/s12936-016-1537-6

**Published:** 2016-09-22

**Authors:** John H. Huber, Geoffrey L. Johnston, Bryan Greenhouse, David L. Smith, T. Alex Perkins

**Affiliations:** 1 Department of Applied and Computational Mathematics and Statistics, University of Notre Dame, Notre Dame, IN USA; 2 Apple Inc., Cupertino, CA USA; 3 Department of Medicine, University of California, San Francisco, CA USA; 4 Institute for Health Metrics and Evaluation, University of Washington, Seattle, WA USA; 5 Department of Biological Sciences and Eck Institute for Global Health, University of Notre Dame, Notre Dame, IN USA

**Keywords:** Epidemiology, Malaria elimination, Mathematical model, Statistical inference

## Abstract

**Background:**

The serial interval is a fundamentally important quantity in infectious disease epidemiology that has numerous applications to inferring patterns of transmission from case data. Many of these applications are *apropos* of efforts to eliminate falciparum malaria from locations throughout the world, yet the serial interval for this disease is poorly understood quantitatively.

**Methods:**

To obtain a quantitative estimate of the serial interval for falciparum malaria, the sum of the components of the falciparum malaria transmission cycle was taken based on a combination of mathematical models and empirical data. During this process, a number of factors were identified that account for substantial variability in the serial interval across different contexts.

**Results:**

Treatment with anti-malarial drugs roughly halves the serial interval due to an abbreviated period of human infectiousness, seasonality results in different serial intervals at different points in the transmission season, and variability in within-host dynamics results in many individuals whose serial intervals do not follow average behaviour. Furthermore, 24.5 % of secondary cases presenting clinically did so prior to the primary cases being identified through active detection of infection.

**Conclusions:**

These results have important implications for epidemiological applications that rely on quantitative estimates of the serial interval of falciparum malaria and other diseases characterized by prolonged infections and complex ecological drivers.

## Background

The basic reproduction number *R*_0_, defined as the expected number of secondary cases arising from a single primary case in a susceptible population, is well known and of fundamental importance in infectious disease epidemiology. Despite extensive efforts to model, measure, and map *R*_0_ globally for falciparum malaria [1, 2], little has been done to quantify its temporal analogue, the serial interval. Defined as the time between the clinical presentation of primary and secondary cases, the serial interval is also of fundamental importance [3]. Probabilistic descriptions of the serial interval provide a basis for identifying sources of infection [4], for assessing whether cases are causally linked [5, 6], for analysing incidence data to estimate temporal variation in transmission and its environmental drivers [7, 8], and for determining whether a pathogen can be declared eliminated [9].

For directly transmitted diseases, the serial interval can be measured through contact tracing or with household data [10, 11]. For malaria and other mosquito-borne diseases, this would require the impossible task of tracing mosquito blood meals between people, so the serial interval must be estimated indirectly. Case data have been analysed with spatiotemporal statistics that are designed to estimate the serial interval by detecting autocorrelation in the appearance of cases (e.g., [12]), but the resolution of these estimates tends to be extremely crude. These statistics are generally not capable of capturing heterogeneity in the serial interval distribution across different contexts, but heterogeneity in the ecology of falciparum malaria across space, time, urban–rural gradients, and in other respects is an important feature of its transmission [13, 14]. An alternative approach [15] with the potential to overcome these shortcomings involves using empirical data to characterize variability in components of the transmission cycle and applying principles of probability to combine those components to describe variability in the length of the transmission cycle as a whole, i.e., the serial interval [3].

For falciparum malaria, one analysis [7] has used such an approach to describe the generation interval, which differs from the serial interval because it pertains to the timing of infection rather than case detection. There are two important limitations of how this approach has been applied to falciparum malaria to date, however. First, applying the generation interval to case data is questionable, given that the generation interval is intended to quantify the timing between infections rather than cases. Second, there are a number of heterogeneities in the falciparum malaria transmission cycle that have not previously been incorporated into descriptions of its generation interval. A more comprehensive quantitative understanding of the falciparum malaria generation interval and serial interval was achieved by considering: (1) differences in the timing of secondary infections arising from asymptomatic or untreated cases as compared with symptomatic cases treated with anti-malarial drugs; (2) variability in entomological parameters that affect the timing of transmission; (3) variability due to seasonal fluctuations in mosquito densities, and (4) inter-individual variability arising from stochastic variation in the trajectory of a given person’s infectiousness over time.

## Methods

### Overview

**Fig. 1:**
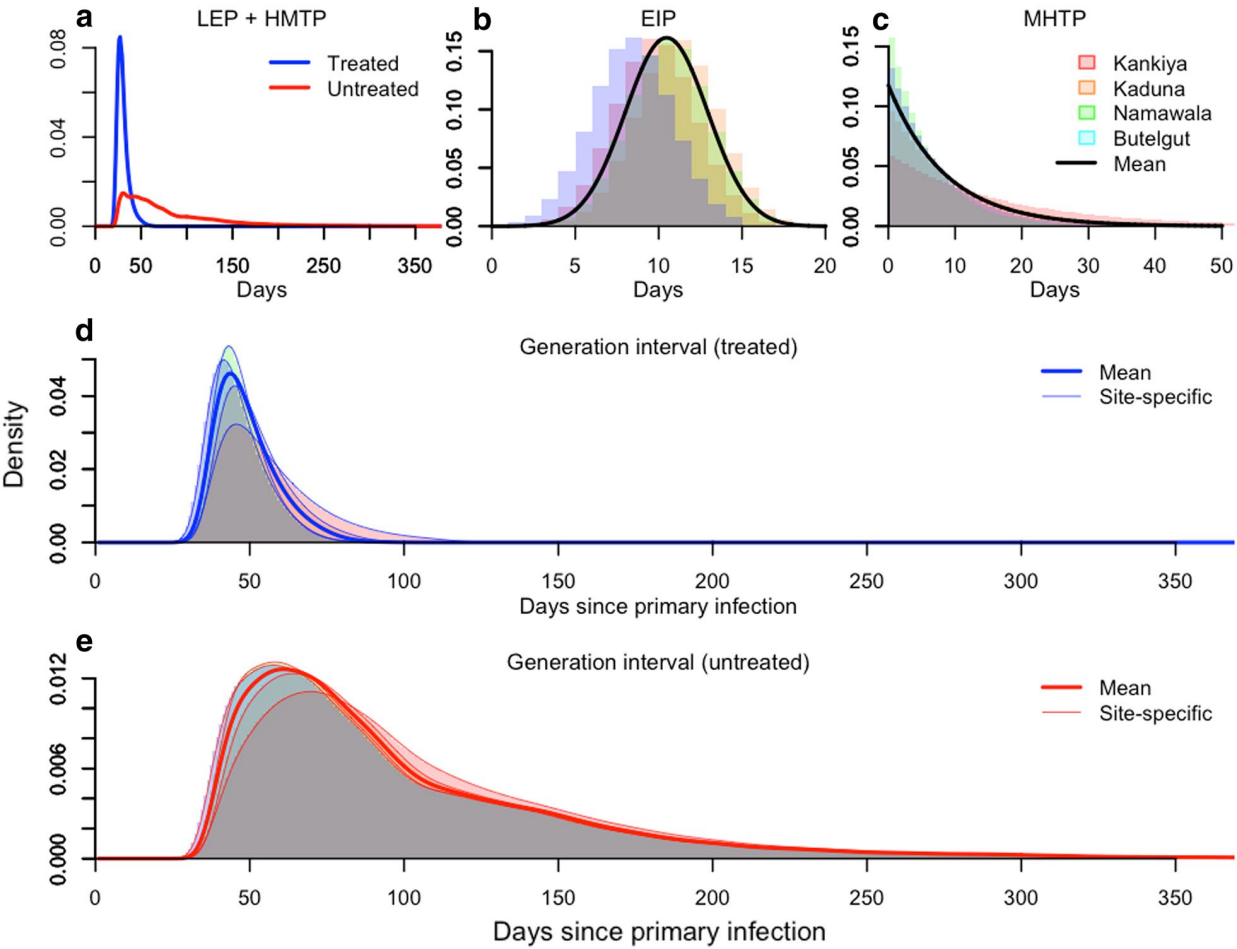
Elements of the falciparum malaria transmission cycle (**a**–**c**) and their impact on variability in the generation interval (**d**, **e**). The first such elements that were delineated were the liver emergence period (LEP) and human-to-mosquito transmission period (HMTP), which differed for primary cases treated with anti-malarial drugs or not (**a**). The third element was the extrinsic incubation period (EIP), whose mean values differed across four representative sites (**b**). The fourth element was the mosquito-to-human transmission period (MHTP), the distribution of which differed for the same four sites due to differences in mean daily mosquito mortalities (**c**). Combining these elements produced four site-specific GI distributions for treated (**d**) and untreated (**e**) primary case scenarios. GI distributions with values of the entomological parameters averaged across sites are shown for comparison in **d** and **e**

To obtain random variables describing the generation interval (GI) and serial interval (SI) of falciparum malaria, random variables were first derived describing components of the GI and SI: the liver emergence period (LEP), the human-to-mosquito transmission period (HMTP), the extrinsic incubation period (EIP), the mosquito-to-human transmission period (MHTP), and the infection-to-detection period (IDP). The exact length of time required for each of these events to occur is inherently random, which is why each must be treated as a random variable rather than as a period of fixed length. Furthermore, because these events must occur sequentially for transmission to occur, the sum of these random variables must be calculated to describe the GI and SI as random variables. The GI is defined as the time between sequential infections, so it is therefore equal to the sum of the LEP, HMTP, EIP, and MHTP (Fig. 1). Because the SI is defined as the time between *detection* of sequential infections, it is defined as the sum of the GI, the IDP for the primary infection, and the IDP for the secondary infection. This approach was applied to quantifying GI and SI distributions under a variety of scenarios to describe variability in the GI and SI across a wide range of conditions typical of falciparum malaria transmission in different settings.

### Probabilistic description of components of the generation and serial intervals

#### Liver emergence period (LEP)

The first period comprising the generation interval for falciparum malaria was defined as the LEP. Consistent with empirical findings [16], this interval between sporozoites entering the skin and asexual merozoites emerging from the liver was modelled as a constant 6 days.

#### Human-to-mosquito transmission period (HMTP)

To simulate the trajectory of blood-stage parasites following their emergence from the liver, a simulation model developed by Johnston et al. [17] was used. This model tracks parasite replication beginning in the first generation after emergence from the liver (e.g., from the sixth day). Once simulated gametocytes were sufficiently mature and abundant to infect mosquitoes [sequestration time~Normal (7 days, 1.5 days)], the probability of a person infecting a blood-feeding mosquito was modelled as a non-linear function of their gametocyte density, consistent with Johnston et al. [17]. Time-varying gametocytaemia and its relationship with infectiousness then governed the infectiousness of a person until the infection was cleared by either the body’s immune response or with the aid of anti-malarial drugs. Because net infectiousness varied substantially across simulations, 1000 replicate gametocytaemia trajectories were generated from Johnston et al. [17, 18] and their average weighted by the net infectiousness of each was computed. This weighted average curve was then normalized to arrive at a probability density describing the HMTP.

The dynamics of gametocytaemia, the immune response, and the effect of anti-malarial drugs were simulated with the model by Johnston et al. [18]. This model was parameterized using malaria therapy data from patients experiencing syphilis, similar to other within-host models of *Plasmodium falciparum* infections in humans [19–22]. These patients had no prior immunity to malaria and were injected with *P. falciparum* in a controlled setting to induce a fever and an immune response. Consequently, the dynamics described by the model by Johnston et al. [17, 18] are most reflective of *P. falciparum* infection dynamics of infections in adult males with no prior exposure. Elaborating on the model by Johnston et al. [17, 18] to better capture the dynamics of infections in individuals with prior exposure and immunity would require data from challenge studies that is not currently available. Thus, this quantification of the HMTP should be regarded as an upper limit, particularly for populations with extensive immunity to *P. falciparum*.

Because the number of mosquitoes blood-feeding on a given day can be highly variable [23], the time-varying probabilities of infection obtained from the Johnston et al. [17] model were multiplied with a potentially time-varying mosquito-to-human ratio, *m*(*t*). The default setting for *m*(*t*) was a constant, but for some analyses a time-varying function was used,

1 $$m(t) = A \phi (t,180,\sigma ) + 1 ,$$

where *A* is peak amplitude, *Φ* is a Normal probability density, and *σ* corresponds to the width of the seasonal peak. To obtain a random variable describing the timing of a mosquito being infected by an infectious human, the time-varying infection probabilities were multiplied by *m*(*t*) and the resulting curve was normalized. The sum of the LEP and HMTP for a constant *m*(*t*) is shown in Fig. 1a.

#### Extrinsic incubation period (EIP)

Once *P. falciparum* gametocytes have been transmitted from an infectious human to a susceptible mosquito, a period of time known as the extrinsic incubation period (EIP) must elapse before sporozoites are produced and disseminated to the mosquito’s salivary glands, where they can then be transmitted to a human. It was assumed that the EIP can be reasonably described by a Normal random variable with mean estimated from any of four sites [24] and standard deviation of 2.47 days, which was estimated based on data digitized from Macdonald [25]. Because the calculations were performed on a daily basis, the EIP was modelled as a random variable with probability mass,

2 $$Pr \; (EIP = i) = \frac{\varPhi (i) - \varPhi (i - 1)}{\varPhi (17) - \varPhi (0)},$$

where *Φ* is a Normal cumulative density (Fig. 1b). By setting the maximum possible EIP at 17 days, Eq. (2) captures 99 % of the total possible EIPs under the distribution of EIP that was considered.

#### Mosquito-to-human transmission period (MHTP)

After a mosquito has become infectious, the final step in the transmission cycle is for the mosquito to transmit parasites to a human. To make this a tractable quantity to model, three simplifying assumptions were made. First, no senescence or any other source of variability in mortality was assumed, such that mosquitoes are subject to a constant daily probability of survival *p*. Second, no assumption about the feeding status of a mosquito at the time it completes the EIP and becomes infectious was made, and the model assumed no correlation between feeding behaviour and lifespan. Third, no effect of mosquito age or time since completion of the EIP on the probability of successfully infecting a human upon blood feeding was assumed. Together, these assumptions imply that the elapsed time between completion of the EIP and the time at which a human is infected can be described as a geometric random variable with probability 1 − *p*. Estimates for the mosquito mortality rate (i.e., 1 − *p*) were taken from Killeen et al. [24]. By setting the maximum possible mosquito lifespan at 30 days beyond completion of the lowest mean EIP that was considered, 99 % of the probability density of this random variable was captured (Fig. 1c).

#### Infection-to-detection period (IDP)

To calculate the serial interval distribution, one additional random variable was defined describing the interval between infection and either presentation at a clinic or detection by other means, such as active surveillance [26]. This interval is referred to as the infection-to-detection period (IDP). IDPs were modelled in different ways for symptomatic (and presumably treated) and asymptomatic (and presumably untreated) cases and are referred to as IDP_S_ and IDP_A_, respectively. Common to both was the interval between sporozoites entering the skin and asexual parasites emerging from the liver, which was assumed to always be 6 days [17].

For symptomatic cases, another random variable was added corresponding to the interval between emergence of parasites from the liver and onset of fever, which was obtained as part of the simulation output of the model by Johnston et al. [18]. The third random variable for symptomatic cases represented time elapsed before seeking treatment some number of days after the onset of fever, which was modelled as a Poisson random variable with parameter λ = 3.07. This value was obtained by maximum-likelihood estimation using data on the timing of treatment seeking relative to fever onset among 1961 falciparum malaria cases from Zanzibar (unpublished data). In combining the IDP for symptomatic cases with the HMTP, the model accounted for the fact that the HMTP is affected by the day on which drugs are administered, because early treatment shortens the duration of human infectiousness to mosquitoes [18]. To account for this, simulations of the HMTP were performed using the model by Johnston et al. [17, 18] with treatment days varying from 0 to 14. Each HMTP was then weighted by its respective IDP_S_ probability and summed to arrive at a combined IDP_S_ + HMTP distribution that accounted for this correlation.

For asymptomatic infections, it was assumed that they were identified through some form of active detection of infection at some point during their infection when their asexual parasitaemia levels exceeded 50 per μL of blood. A probability distribution describing the probability that such a level of asexual parasitaemia exceeded this threshold on a given day was obtained by directly calculating the empirical density of the number of days in excess of 50 per μL from 1000 realizations of the simulation model by Johnston et al. [17]. Equating IDP_A_ with this distribution assumes that active detection of infection is attempted only once during the infection and that its timing is random with respect to day since infection. Such would be the case in the context of reactive case detection or during a single cross-sectional survey, for example. In the event that active detection occurs at some regular interval less than the total period of infection, IDP_A_ would be shorter. Although this may be important for accurately characterizing IDP_A_ in certain surveillance contexts, the impact on IDP_A_ of these and potentially many other variations on surveillance systems are beyond the scope of this work.

### Calculation of generation and serial intervals

To obtain a probabilistic description of the generation interval, the LEP, HMTP, EIP, and MHTP random variables were summed by direct convolution, resulting in

3 $$\begin{aligned} &{\text{GI}} \, (i +j + k + l ) \\ &= \sum\limits_{i} \sum\limits_{j}\sum\limits_{k}\sum\limits_{l} {(\Pr ({\text{LEP}} = i) \, { \times }} \, \Pr ({\text{HMTP}} = j) \\ & \quad { \times }\Pr ({\text{EIP}} = k) \, { \times }\, \Pr ({\text{MHTP }} = l)) \end{aligned}$$

where *i*, *j*, *k*, and *l* are dummy variables. In other words, Eq. (3) calculates the probability that *i*, *j*, *k*, and *l* sum to a particular value of the GI for all possible combinations of *i*, *j*, *k*, and *l* that would be compatible with a given sum GI = *i* + *j* + *k* + *l*. These different combinations are furthermore weighted in Eq. (3) by the respective probabilities that each component of the GI takes a given value of *i*, *j*, *k*, or *l*. Similarly, to obtain a probabilistic description of the serial interval, the GI and IDP random variables were summed, also by direct convolution, resulting in

4 $$\begin{aligned} &{\text{SI}}\left( { - i + j + k} \right) \\ & = \mathop \sum \limits_{i} \mathop \sum \limits_{j} \mathop \sum \limits_{k} \left( {\Pr \left( {{\text{IDP}} = i} \right) \times \Pr \left( {{\text{GI}} = j} \right) \times \Pr \left( {{\text{IDP}} = k} \right)} \right). \end{aligned}$$

Note that the IDP appears in Eq. (4) twice because one instance corresponds to the primary infection and the other corresponds to the secondary infection.

### Sources of variability in generation and serial intervals

Using this framework, variability in GI distributions that arises from variability in model parameters in several ecological and epidemiological contexts was quantified. The analysis of variability in SI distributions was limited to the effects of anti-malarial drugs, given that treatment affects both components of the SI, i.e., the GI and the IDP. Other sources of variability in GI distributions, such as geographical, seasonal, and inter-individual variability, are likely to generate similar variability in SI distributions. For both GI and SI distributions, the mean and fifth and 95th percentiles were routinely calculated and reported, as they can be readily calculated numerically.

#### Variability between individuals treated with anti-malarial drugs or not

To address impacts of treatment with anti-malarial drugs on the generation interval of falciparum malaria, the model by Johnston et al. [17, 18] was simulated to obtain human infectivity trajectories assuming no drug treatment and assuming a standard regimen of treatment with artemisinin-based combination therapy (ACT). Treatment with ACT was modelled according to default settings in Johnston et al. [18]. In order to address variation in the lag between the manifestation of symptoms and the start of treatment, the day between the onset of fever and clinical presentation was varied from zero to 14 days, where a delay of zero days signified that the individual presented in the clinic the same day that the fever manifested. It was assumed that clinical presentation marked the first day of administration of anti-malarial drugs. These infectivity curves were then weighted with their respective probabilities from the Poisson distribution describing the time elapsed between fever onset and clinical presentation to arrive at a mean infectivity curve for individuals treated with anti-malarial drugs.

#### Geographic variability in entomological indices

To account for variability in entomological parameters, the GI distribution was calculated under four different parameterizations of the mean EIP and daily probability of mosquito mortality corresponding to four different sites, as reported by Killeen et al. [24]. These sites and parameter values were: Kankiya, Nigeria (mean EIP = 10.3, daily mortality = 0.06); Kaduna, Nigeria (11.6, 0.10); Namawala, Tanzania (11.1, 0.17); and Butelgut, Papua New Guinea (8.9, 0.14). In cases where two estimates were reported by Killeen et al. [24], their average was used in the calculations.

#### Seasonal fluctuations in mosquito densities

To determine the extent to which seasonal fluctuations in mosquito densities could introduce variability into the GI distribution, *m*(t) was set equal to the time-varying function in Eq. (1) and used to calculate the resulting GI and SI distributions. These calculations were performed under four scenarios about the parameters in Eq. (1): *A* = 1, *σ* = 14; *A* = 9, *σ* = 14; *A* = 1, *σ* = 120; and *A* = 9, *σ* = 120. Values of *A* equal to 1 and 9 led to two- or ten-fold increases, respectively, in the ratio of high-season to low-season mosquito densities. Values of *σ* of 14 or 120 lead to narrow or wide seasonal peaks in mosquito density, respectively.

#### Inter-individual variability in gametocytaemia trajectories

To assess the extent of possible variability in different GI and SI distributions among different individuals with different realized HMTP distributions, 1000 unique realizations of HMTP distributions were simulated from the model by Johnston et al. under default settings [17, 18]. These 1000 distributions were compared against the default HMTP distribution, which was obtained by computing the mean across these 1000 replicate distributions at each given day since the beginning of the HMTP.

## Results

### Variability between individuals treated with anti-malarial drugs or not

The overall shape of the GI distribution was dependent on the status of the primary infection with respect to anti-malarial drug treatment, with mean (5–95 percentile) GIs of 49.1 (35.0–68.0) and 101.6 (43.0–219.0) days for treated and untreated primary infections, respectively (Fig. 1d, e). Overall, GIs arising from untreated primary infections were much longer, with 95 % of secondary infections being infected by day 68 for treated primary infections as opposed to 219 days for untreated primary infections.

**Fig. 2:**
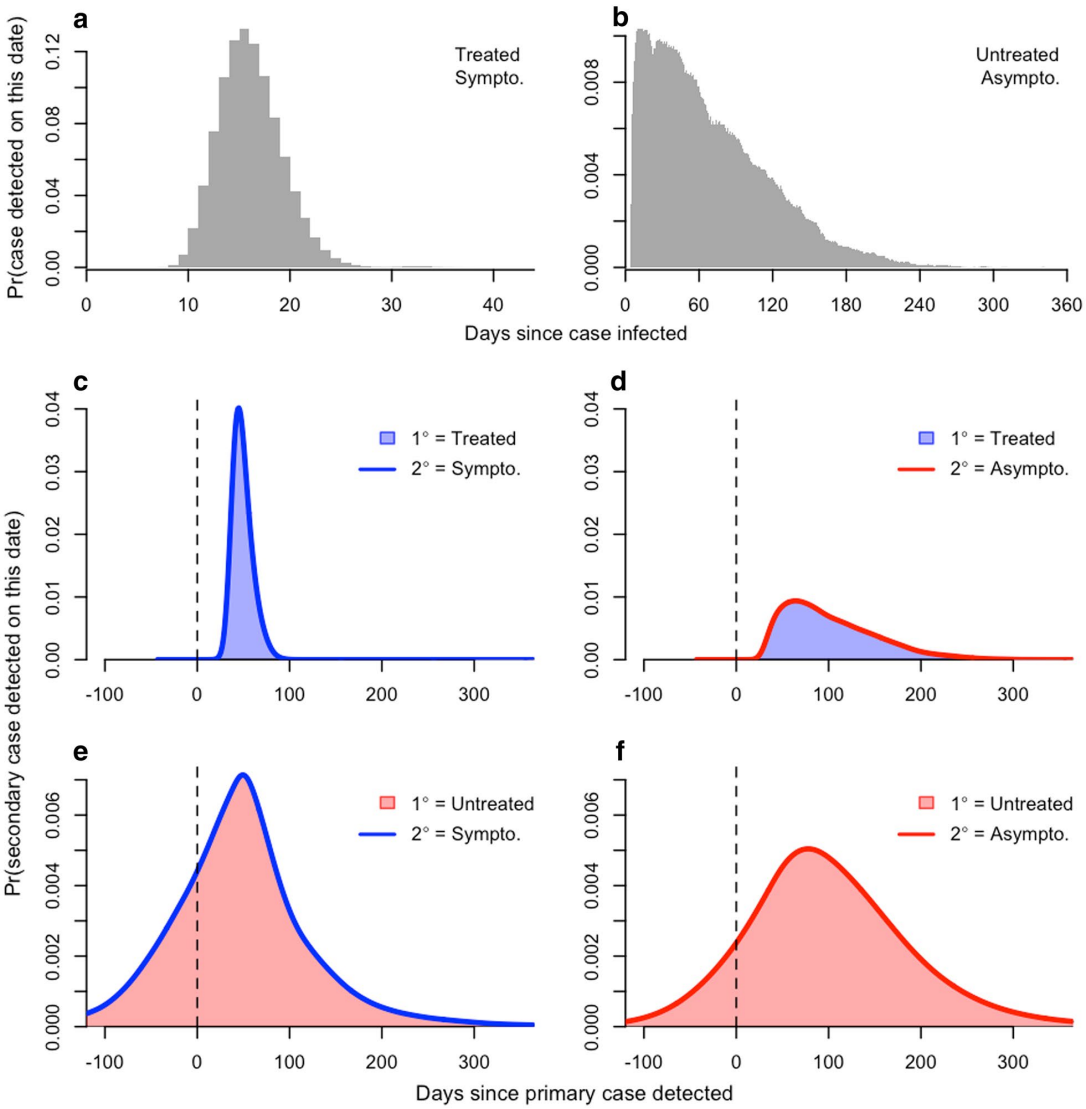
Probabilistic descriptions of the infection-to-detection periods IDP_S_ and IDP_A_ between infection and detection of treated/symptomatic (**a**) and untreated/asymptomatic (**b**) cases, respectively. The SI random variable is obtained by summing the GI random variable with random variables describing the IDP once for the primary infection and once again for the secondary infection. Combining IDP random variables with the appropriate generation interval random variables yielded four different estimates of the serial interval: treated primary case and either symptomatic (**c**) or asymptomatic (**d**) secondary case; untreated primary case and either symptomatic (**e**) or asymptomatic secondary case (**f**)

**Fig. 3:**
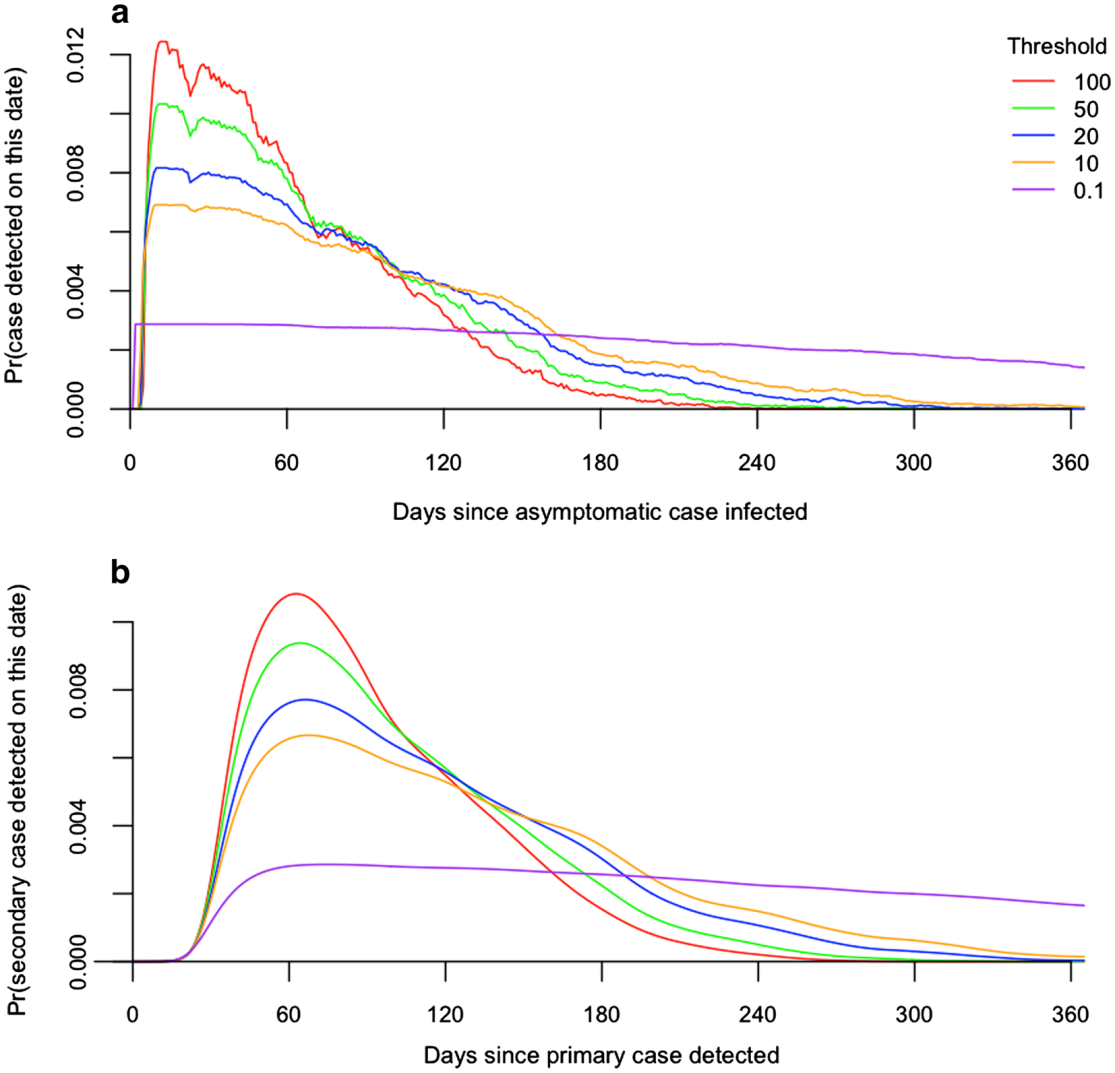
Variability associated with differences in the asexual parasitaemia detection threshold for a secondary infection detected by active detection of infection. Panel **a** shows normalized probability densities for the infection-to-detection period IDP_A_ as a function of the detection threshold (asexual parasites per μL of blood). Panel **b** shows normalized probability densities for the SI given an untreated primary case as a function of the corresponding IDP_A_ distributions from **a**

SI distributions were formed by combining the generation interval distributions with the period between the time of infection by a mosquito and the detection of the infection for both the primary and secondary infections. This IDP for symptomatic infections, IDP_S_, was relatively short, with mean (5–95 %) of 16.6 (12.0–22.0) days (Fig. 2a). The IDP for asymptomatic infections, IDP_A_, was relatively long and fat-tailed, with mean (5–95 %) of 69.8 (11.0–163.0) days (Fig. 2a). The IDP_A_ distribution was also somewhat sensitive to the choice of the asexual parasitaemia threshold for detection (Fig. 3).

Combining GI and IDP distributions showed that there are four different types of SI distributions, all with somewhat different features (Fig. 2). For a primary infection that is symptomatic and presumably treated with drugs, secondary infections that are also symptomatic would be expected to appear 49.1 (33.0–69.0) days after detection of the primary infection (Fig. 2c). Secondary infections that are asymptomatic would be expected to appear 102.2 (33.0–197.0) days after detection of a primary symptomatic infection (Fig. 2d), assuming that the infections are detected at all. For a primary infection that is asymptomatic and presumably only detected in the context of an active epidemiological investigation, secondary infections that are symptomatic would be expected to appear 48.4 (−70.0 to 181.0) days after detection of the primary infection (Fig. 2e). Secondary infections that are asymptomatic would be expected to appear 101.6 (−36.0 to 261.0) days after detection of a primary asymptomatic infection (Fig. 2f), assuming that either is detected at all. Of secondary infections that present clinically, 24.5 % are expected to be detected before the associated primary infection is detected, even in the event of active detection of infection during an epidemiological investigation (Fig. 2e). When both primary and secondary infections are asymptomatic and detected through active detection of infection, 11.5 % of secondary infections could be detected prior to detection of the primary infection (Fig. 2f), assuming that both are detected at all.

### Geographic variability in entomological indices

Using entomological parameters from four diverse sites (Fig. 1b, c), means and 5–95 percentiles of the GI distributions varied from 46.1 (33.0–63.0) to 56.6 (37.0–89.0) for treated primary infections (Fig. 1d) and from 98.7 (41.0–216.0) to 109.2 (46.0–228.0) for untreated primary infections in Butelgut and Kankiya, respectively (Fig. 1e). By comparison, the modes of the GI distributions ranged 42–46 for treated primary infections and 58–70 for untreated primary infections (Fig. 1d, e). The long GI for Kankiya appears to be driven mostly by relatively low mosquito mortality, and the short GI at Butelgut appears to be driven by both a short EIP and relatively high mosquito mortality (Fig. 1b, c).

### Seasonal fluctuations in mosquito densities

**Fig. 4:**
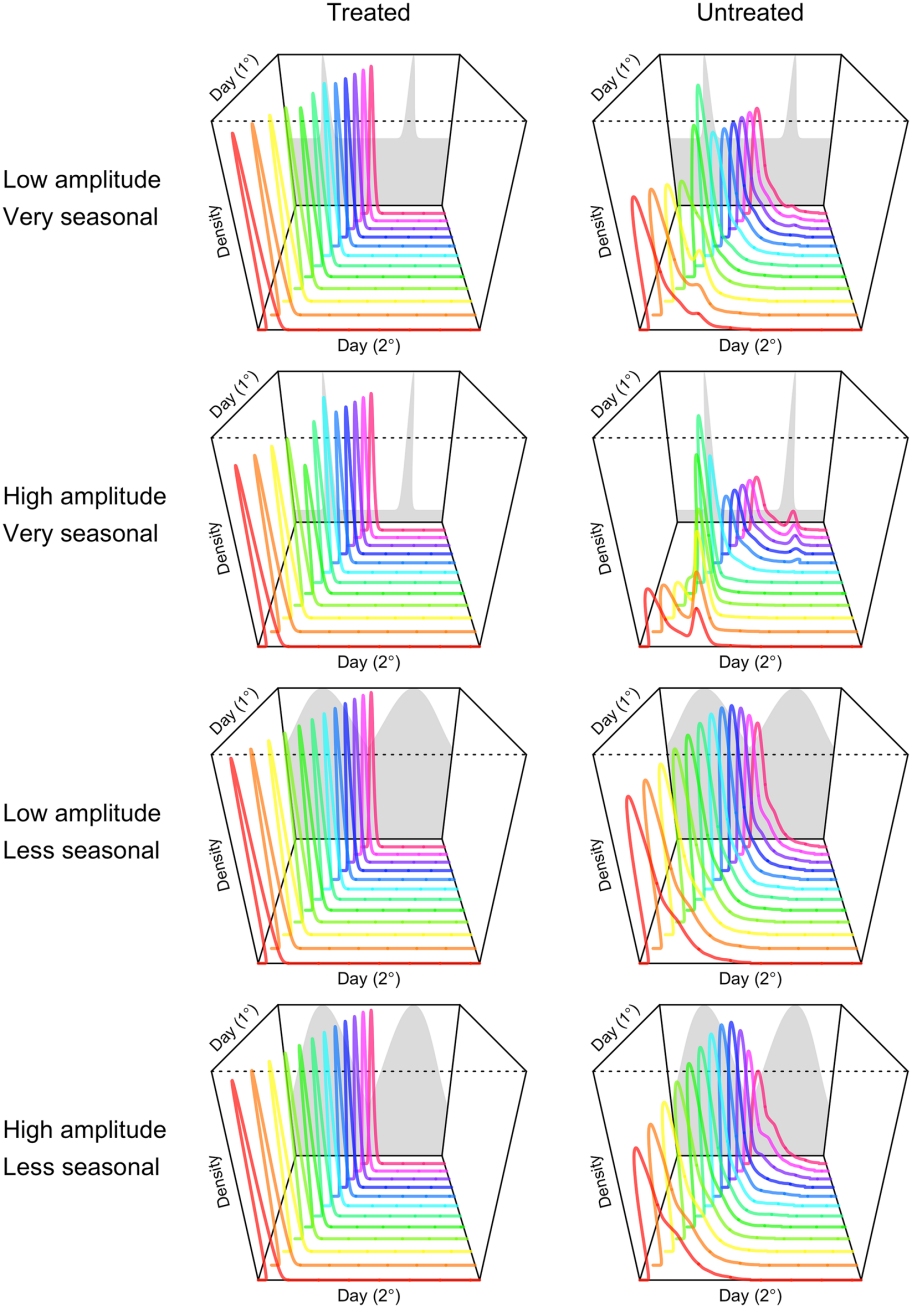
Variability in the generation interval distribution in a seasonal environment for treated and untreated primary cases (*columns*) in seasonal environments with different properties (*rows*). Seasonality was imposed by forcing mosquito densities consistent with the grey shapes in the background of each panel, which vary in their amplitude and the distinctiveness of the seasonal peak at day 180 in each of 2 years. SI distributions are shown for primary infections occurring on days 1 through 360 in increments of 30

Differences in GI distributions owing to differences in timing relative to a seasonal transmission peak were more substantial, affecting not only the moments of the GI distribution but also its shape (Fig. 4). These effects were most pronounced for untreated primary infections, whose GI distributions spanned a broader portion of the year (Fig. 4, right column). Primary infections timed well before the seasonal peak tended to be associated with more secondary infections later than they would have in a constant environment, and the peak of the GI distribution for primary infections timed just before the seasonal peak tended to be more peaked and narrower than it would have been otherwise. The extent of these differences depended on the extent to which transmission was seasonally peaked (Fig. 4, second row).

### Inter-individual variability in gametocytaemia trajectories

**Fig. 5:**
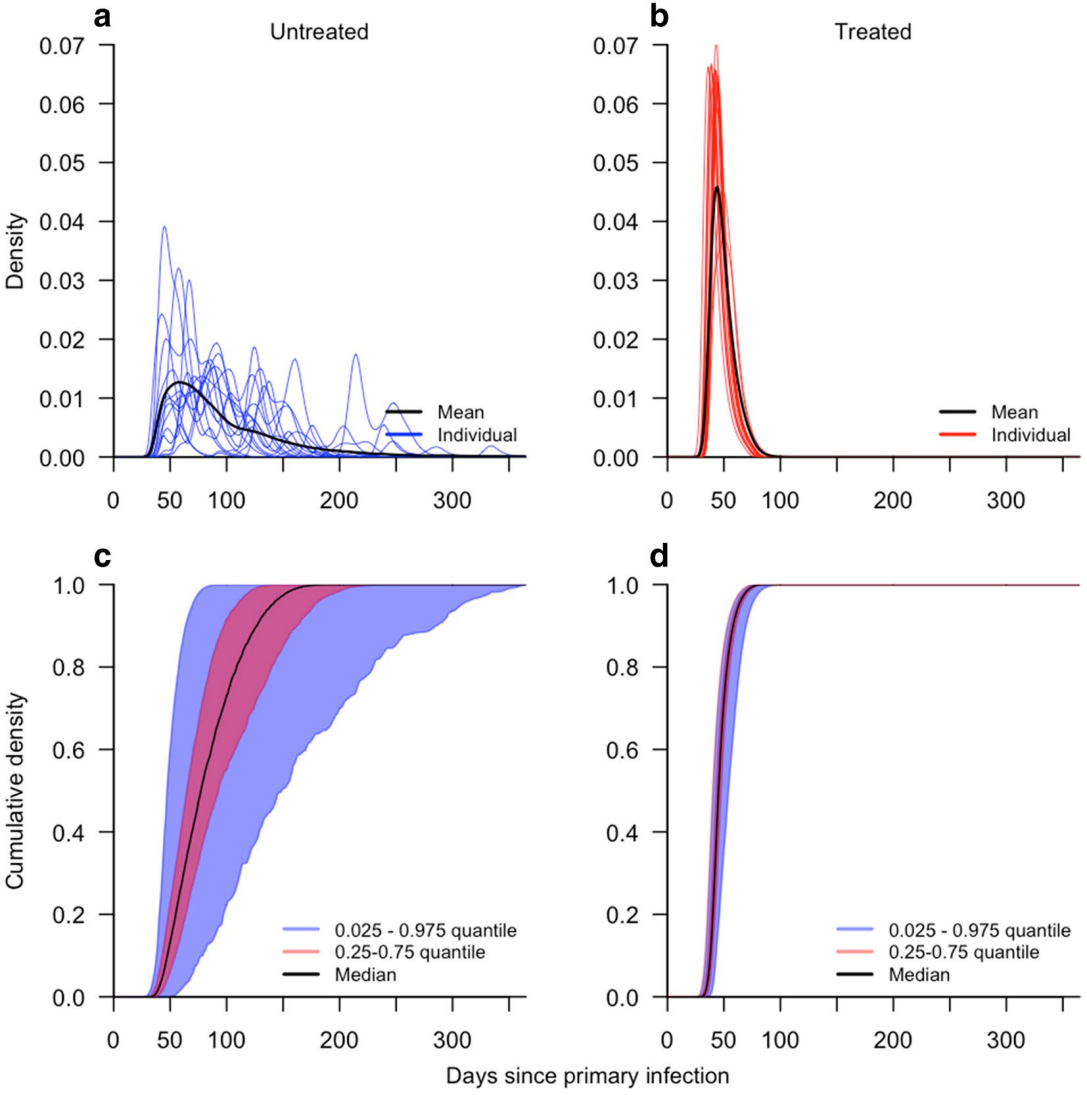
Variability in generation interval distributions ensuing from primary infections that do (**b**, **d**) or do not (**a**, **c**) receive anti-malarial drugs. **a** and **b** show normalized probability densities for mean and representative GI distributions from 15 realizations of the simulation model. **c** and **d** show quantiles of cumulative probability densities for 1000 realizations of the simulation model

The final source of variability in the GI distributions that was examined pertained to variability in the timing of infectiousness of humans to mosquitoes across 1000 simulated primary infections with the same drug treatment status. For primary infections receiving anti-malarial drugs, the distributions of the HMTP across different individuals were relatively uniform, with all individual trajectories rising and falling relatively quickly (Fig. 5a, b). For primary infections not receiving anti-malarial drugs, distributions of the HMTP across different individuals were much more variable. Unlike treated infections, untreated infections displayed simulated HMTP distributions with multiple peaks; the number, timing, and height of which varied considerably (Fig. 5c). These differences lead to broad variability in quantiles of the GI distribution. For example, the median GI varied by over 100 days for the inner 95 % of individual GI distributions.

## Discussion

One of the first attempts to quantify the serial interval for falciparum malaria was by Macdonald [27], who posited that 36 days represents a minimum estimate based on hard biological constraints. Much more recently, Churcher et al. [7] posited a mean generation interval of 102 days for individuals who do not receive anti-malarial drug treatment and 33 days for those who do. The estimates presented here are in good agreement with the former (102 days) but not the latter (48 days). One reason for the nearly 50 % increase in the estimate of the mean GI for treated primary infections relative to that by Chrucher et al. [7] has to do with differences in the assumptions about the delay between onset of symptoms and seeking of treatment. Although their means were nearly identical, the exponential distribution used by Churcher et al. [7] resulted in appreciably more individuals seeking treatment on the same day as symptom onset than the Poisson distribution used in this study did. Collectively, this and other seemingly subtle differences led to a large discrepancy between the means of this study’s and Churcher et al.’s [7] distributions for treated infections. The discrepancy between the distributions for untreated infections was smaller due to the dominance of a much lengthier period of human infectiousness.

An increasingly important application of probabilistic descriptions of the SI is the inference of transmission linkages between cases [5–7, 28]. Given the breadth of the SI distributions described here, using temporal information alone to link falciparum malaria cases may be inadvisable. First, even in the best-case scenario of a putative transmission linkage between two known cases that promptly sought treatment, the SI distribution is sufficiently wide that distinguishing that linkage from others within a time period of a few weeks should be largely uninformed by temporal data alone. Second, the shape of the GI distribution differs considerably from person to person due to the complex within-host dynamics of *P. falciparum* infections [6, 29]. This may preclude the inference of transmission linkages ensuing from primary cases whose infections do not follow average behaviour. Third, SI distributions associated with cases that were or were not treated with anti-malarial drugs differ substantially, with the latter even being negative in many cases (i.e., the secondary case is detected before the primary case is detected). Given that negative values are strictly impossible for GIs, this underscores the importance of being conscientious about the distinction between GIs and SIs when applying these methods to case data (as in [6]).

Probabilistic descriptions of GIs and SIs also have an important role to play in population-level models of infectious disease dynamics. Together with an estimate of epidemic growth rate, the GI distribution can be used to estimate the basic reproduction number and related quantities [8, 30, 31]. Any time that there are secular changes in factors that affect transmission within the timeframe of a single generation, however, there is a risk of being misled by a static description of the GI or SI distribution. Similar to the analysis herein of how seasonally varying mosquito densities affect the falciparum malaria GI, an analysis by Vynnycky and Fine [32] showed that not accounting for secular trends in contact rates over time led to an underestimate of tuberculosis transmission potential. This result underscores the conclusion that there is no one-size-fits-all description of GIs and SIs, particularly for long-lasting infections such as falciparum malaria.

## Conclusions

The study has highlighted a number of reasons why GI and SI distributions are variable for falciparum malaria and offered quantitative remedies to modelling many of these situations. To this end, code for reproducing the figures and for calculating GI and SI distributions over one or more generations of falciparum malaria cases is available online [33]. Like many topics in epidemiology, robust quantification of GI and SI distributions stands to benefit from careful and empirically well-grounded use of mechanistic models to describe constituent processes in the transmission cycle.

## Abbreviations

ACT: artemisinin-based combination therapy
EIP: extrinsic incubation period
GI: generation interval
HMTP: human-to-mosquito transmission period
IDP: infection-to-detection period
LEP: liver emergence period
MHTP: mosquito-to-human transmission period
SI: serial interval
